# Evaluation of Sarcopenia, Frailty, and Inflammation on Adverse Events and Survival Outcomes in Patients with Oral Cavity Squamous Cell Carcinoma under Adjuvant Chemoradiotherapy

**DOI:** 10.3390/jpm11090936

**Published:** 2021-09-19

**Authors:** Chun-Hou Huang, Peir-Rorg Chen, Kun-Han Lue, Tsung-Cheng Hsieh, Yu-Fu Chou

**Affiliations:** 1 Department of Nursing, Tzu Chi University, Hualien 97004, Taiwan; hou2017@gms.tcu.edu.tw; 2 Department of Otolaryngology, Hualien Tzu Chi Hospital, Buddhist Tzu Chi Medical Foundation, Hualien 97004, Taiwan; cprong@gms.tcu.edu.tw; 3 School of Medicine, Tzu Chi University, Hualien 97004, Taiwan; 4 Department of Medical Imaging and Radiological Sciences, Tzu Chi University of Science and Technology, Hualien 97005, Taiwan; john.lue@protonmail.com; 5 Institute of Medical Sciences, Tzu Chi University, Hualien 97004, Taiwan; tchsieh@gms.tcu.edu.tw

**Keywords:** sarcopenia, frailty, platelet/lymphocyte ratio, oral cavity squamous cell carcinoma

## Abstract

In this study, we aimed to evaluate the prognostic impact of sarcopenia, five-item modified frailty index (mFI-5), neutrophil/lymphocyte ratio (NLR), and platelet/lymphocyte ratio (PLR) in patients with oral cavity squamous cell carcinoma (OSCC) treated with adjuvant chemoradiotherapy (CRT) and their survival outcomes. We retrospectively enrolled 175 patients with OSCC undergoing adjuvant CRT between 2011 and 2018, who were divided into groups with (*n* = 112) and without (*n* = 63) sarcopenia. Logistic regression analysis and Cox proportional hazards models were used to determine prognostic factors for CRT-related toxicity, three-year overall survival (OS), and disease-free survival (DFS). Sarcopenia and high PLR were independently associated with CRT-induced anemia (CIA); advanced tumor stage was related to poor three-year OS. CRT and survival did not differ by mFI-5 and NLR. Our results indicate that sarcopenia and high PLR are significant predictors of adjuvant CRT, increasing toxicity outcomes and indicating worse short-term OS. Accurately identifying sarcopenia and high PLR in patients with OSCC is critical to help better select candidates for adjuvant CRT to improve their outcomes.

## 1. Introduction

Oral cavity squamous cell carcinoma (OSCC) was a major cause of cancer death worldwide in 2018 [1]. The primary treatment is radical resection, followed by radiotherapy or chemoradiotherapy (CRT) [2]. Current precision therapies include optimizing radiation, targeted therapies, immunotherapy, and gene therapy. Unfortunately, the prognosis and quality of life remain low [3,4]. Almost 70% of patients with head and neck cancer (HNC) present at diagnosis with advanced disease, often accompanied by comorbidities and nutritional deficiencies. The patients may also develop sarcopenia, which is multifactorial, with physical inactivity, systemic inflammation, increased metabolic rate, and reduced nutrient intake [5,6].

Up to 65% of patients with HNC developed sarcopenia during the pretreatment period [6]. In patients with HNC, sarcopenia is independently associated with poor outcomes [7,8,9,10,11,12], such as dose-limiting chemotherapy toxicities [9], higher risk of postoperative AEs [10,11,12], and lower progression-free and overall survival (OS) [8,11,12]. Therefore, predicting short-term clinical outcomes in patients with OSCC is essential. Systemic inflammation-based blood leukocyte indices, including the neutrophil/lymphocyte ratio (NLR) and the platelet/lymphocyte ratio (PLR), are strongly correlated with cancer prognosis. Both reflect tumor-promoting activities, including angiogenesis, mutagenesis, and immunosuppression [13]. The association between raised PLR or NLR and worse prognosis was observed in HNC [14], although these studies were heterogeneous for clinical characteristics or different treatment modalities.

Frailty implies a state of heightened vulnerability to acute and chronic stressors caused by a significant reduction in physiologic reserves, increasing the risk of poor clinical outcomes [15]. While sarcopenia is a precursor of frailty, these two are not interchangeable [16]. In recent years, frailty evaluations have been increasingly used to predict perioperative mortality and morbidity in patients with HNC [17,18,19,20,21]. Although extensively studied in patients undergoing surgery, there are less data on patients with OSCC exposed to CRT stressors. Therefore, our study aims to examine the predictive role of sarcopenia, frailty status, NLR, and PLR on CRT-related toxicities and survival outcomes in patients with OSCC.

## 2. Materials and Methods

### 2.1. Patients and Study Design

This retrospective study was approved by the Institutional Review Board and Ethical Committee of Hualien Tzu Chi General Hospital, Buddhist Tzu Chi Medical Foundation (IRB no.: IRB109-292-B). We included patients with OSCC who underwent pre-CRT head and neck computed tomography (CT), and who were treated at our institution between 2011 and 2018 with curative intent surgery, followed by adjuvant CRT. Exclusion criteria were as follows: (1) previous history of malignancy, (2) only surgery or only radiotherapy or supportive care, and (3) lost to follow-up or had incomplete data. All patients who received adjuvant CRT were administered a total prescribed dose of 60–66 Gy to the tumor bed with the dissected nodal region and a prophylactic dose of 54–60 Gy to the undissected neck nodal region at risk [22]. Chemotherapy combined with platinum-based chemotherapy plus +/− oral tegafur-uracil or +/− 5-FU infusion as indicated depends on several risk factors, i.e., positive margins, extracapsular spread (ECS), lymphovascular invasion (LVI), perineural invasion (PNI), close margins, and depth of invasion [2,3].

From the electronic chart review, we collected age, gender, smoking and alcohol history, five-item modified frailty index (mFI-5), cancer site, clinical stage, clinicopathologic characteristics (PNI, LVI, ECS), nutritional status (serum albumin), body mass index (BMI), and skeletal muscle index (SMI). In routine blood tests, white blood cell count, hemoglobin (Hb), platelet count, and differential white blood cell count were examined. The laboratory examination of blood AEs was only assessed because symptomatic toxicity might have been overlooked in the review of the medical records. The laboratory data were collected within one week post-chemotherapy and radiotherapy. Hematologic AEs were graded according to the National Cancer Institute Common Terminology Criteria for Adverse Events, v.5.0 [23]. Survival analysis was performed for three-year OS (calculated from the date of diagnosis to the date of death or censored at the date of three years of follow-up for surviving patients) and disease-free survival (DFS; the time between the end of diagnosis and the date of recurrence or the date of death or censoring at three years of follow-up).

### 2.2. Definition of Sarcopenia, Frailty, and Inflammatory Status

**Figure 1:**
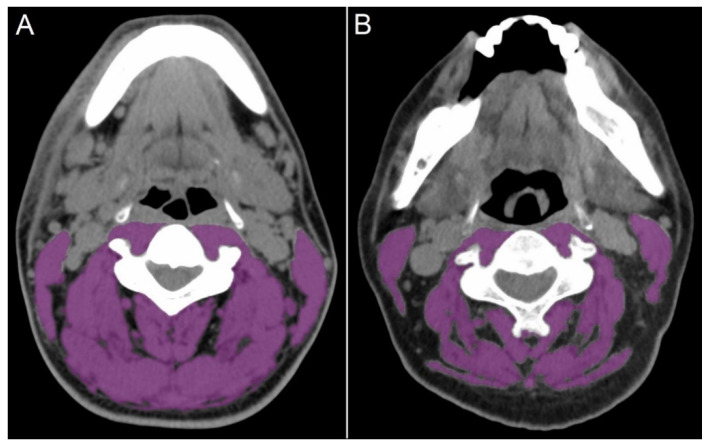
Skeletal muscle area regions in computed tomography images at the third cervical vertebral level in patients without (**A**) and with (**B**) sarcopenia.

Sarcopenia was determined based on a single-slide CT measurement of the cross-sectional skeletal muscle area (SMA) at the level of the third lumbar vertebra (L3) [24,25]. The SMA at the third cervical vertebra (C3) level can be converted to SMA at the level of L3 using a formula published by Swartz et al. [25]. The SMA of all patients was measured at the C3 level on the CT images before surgery. A single axial CT slice at C3 showing the entire vertebral arc was selected. The SMA was quantified at the slice by applying a threshold within −29 to +150 Hounsfield units [25,26,27]. The SMA (cm^2^) at C3 was converted using the equation described by Swartz et al. [25] to estimate the SMA at L3. This value was then adjusted for the patient’s height (m^2^), resulting in the lumbar SMI (cm^2^/m^2^) to define sarcopenia [24,25,26,27]. All images were analyzed using the open-source software OsiriX (Pixmeo, Geneva, Switzerland) [28]. Sarcopenia was defined using previously determined thresholds of an SMI below 46.7 cm^2^/m^2^ for men and 30.3 cm^2^/m^2^ for women [12]. Figure 1 shows the C3 slices on the CT images of patients without (A) and with (B) sarcopenia.

Frailty was quantified using the mFI-5, a modified form of the 11-item frailty index based on the Canadian Study of Health and Aging Frailty Index that is used to quantify a series of “accumulating deficits” often seen in elderly patients [29]. The mFI-5 variables are consistently recorded and verified in the National Surgical Quality Improvement Program dataset to assess frailty in surgical specialties [30,31,32]. The index results in a score (0–5) where 1 point each is attributed to having a history of chronic obstructive pulmonary disease, congestive heart failure, diabetes mellitus, hypertension requiring medication, and functional dependency (Eastern Cooperative Oncology Group ≥ 3). Based on previous literature [30], we stratified the patients by mFI-5 score into 0 (no comorbidities), 1 (1 comorbidity, defined as pre-frailty), and 2+ (2 or more comorbidities, defined as frailty).

The inflammatory status was evaluated before adjuvant CRT treatment with hematologic markers NLR and PLR. NLR and PLR were calculated as the ratio of neutrophil cell and platelet counts to lymphocyte cell counts, respectively. The optimal cutoff values for NLR and PLR were determined using receiver operating characteristic (ROC) curve analysis based on Youden’s index [33].

### 2.3. Statistical Analysis

Data were analyzed using the SPSS statistical package v.20 (SPSS, Chicago, IL, USA). Descriptive statistics such as frequency, percentage, mean, and standard deviation (SD) or the median and interquartile range (IQR) were provided for the sarcopenia and non-sarcopenia groups. Independent *t*-tests or Mann–Whitney U tests were used for continuous variables. The Chi-square or Fisher’s exact test was performed for comparisons between two groups for categorical variables. Logistic regression models were used to evaluate the association between CRT toxicity and sarcopenia (yes or no). Survival curves were obtained using the Kaplan–Meier method, and the log-rank test was used for comparison. Univariate and multivariate analyses of CRT toxicities and three-year OS and DFS were performed using the Cox proportional hazards model, and the results are presented as hazard ratio (HR) with 95% confidence interval (CI). *p* value < 0.05 was considered statistically significant.

## 3. Results

### 3.1. Patient Characteristics

We performed a review of the medical records of 267 patients who received adjuvant treatment. Patients were excluded if: (1) preoperative CT images could not be obtained (*n* = 36); (2) they had incomplete or missing data (*n* = 65). Thus, 175 patients were identified to be eligible for inclusion in this study. No significant differences in characteristics were noted between patients who were included and those who were not (data not shown). All tumors were histologically confirmed to be OSCC. Sarcopenia was identified using pre-CRT imaging in 112 patients (64%). The presence of sarcopenia was associated with being male (*n* = 112, 100%), being a former or current alcohol drinker (*n* = 93, 83.1%), a higher mFI-5 score (≥2: *n* = 23, 20.6%), a lower BMI (23.6 ± 3.4 kg/m^2^), and a lower SMI (38.8 ± 5.7 cm^2^/m^2^). There were no significant differences in age, prevalence of smoking and betel nut chewing, tumor characteristics, and pretreatment hematologic markers between the groups (Table 1). The ROC curve was constructed for NLR and PLR with three-year OS as the primary endpoint. A high PLR was defined as >385, with an area under the curve of 0.66, a sensitivity of 56.2%, and a specificity of 74%. Since there was no significant ROC curve to determine the optimal cutoff value of NLR, we used the median value of 3.4. 

### 3.2. Hematologic Toxicities under CRT According to Sarcopenia and Frailty Status

For hematologic toxicities, anemia significantly occurred more often in the sarcopenia group (17.9%) than in the non-sarcopenia group (6.4%; *p* = 0.021) (Table 2). In terms of grade 3 and 4 toxicities, febrile neutropenia (FN) and infection were not significantly different between the groups. Baseline characteristics were not significantly different between groups stratified by frailty status (data not shown). In the patients with or without frailty with AEs, there were no significant differences in anemia, FN, or infection after CRT. Logistic regression analysis of baseline clinical variables for CRT-related toxicities showed that the independent predictors of anemia (≥grand 3) were sarcopenia (odds ratio (OR) = 1.871, 95% CI = 1.051–3.320) and high PLR (OR = 3.102, 95% CI = 1.017–8.912). In patients ≥65 years old, advanced tumor stage (stage III–IV), sarcopenia, lower Hb, and high NLR/PLR were not significantly associated with FN and infection, as shown in Table 3.

### 3.3. Survival Analysis

**Figure 2:**
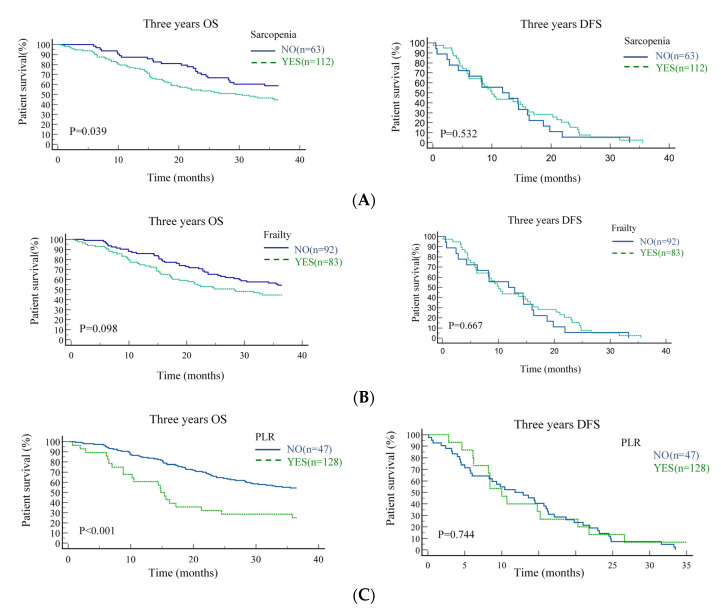
Kaplan–Meier estimates of the three-year overall survival and disease-free survival according to the presence of sarcopenia (**A**), frailty status (**B**), and platelet/lymphocyte ratio (**C**).

The median follow-up was 83 months (95% CI = 38.5–129.1). The results of univariate and multivariate Cox regression analyses for the clinical variables on three-year OS are presented in Table 4. In the univariate analysis, advanced tumor stage (HR = 2.352, 95% CI = 1.415–3.180), positive PNI (HR = 1.631, 95% CI = 1.104–2.423), positive ECS (HR = 1.727, 95% CI = 1.122–2.624), sarcopenia (HR = 1.745, 95% CI = 1.123–2.627), high NLR (HR = 1.793, 95% CI = 1.116–2.176), and high PLR (HR = 2.620, 95% CI = 1.610–4.213) were associated with three-year OS. These variables were tested in the multivariate Cox regression model. After multivariate analysis, advanced tumor stage (HR = 1.820, 95% CI = 1.201–3.323), sarcopenia (HR = 1.643, 95% CI = 1.202–2.670), and high PLR (HR = 1.983, 95% CI = 1.206–2.553) maintained their prognostic significance for three-year OS. Kaplan–Meier analysis was performed for patients with and without sarcopenia and by frailty status and PLR. Three-year OS was significantly better in patients without sarcopenia (*p* = 0.039); meanwhile, DFS was not significantly different between the groups (*p* = 0.532) (Figure 2A). Patients with or without frailty had no significant difference in three-year OS (*p* = 0.098) or DFS (*p* = 0.667) (Figure 2B). Three-year OS was significantly better in patients with low PLR (*p* < 0.001); DFS was not significantly different between the groups (*p* = 0.744) (Figure 2C).

## 4. Discussion

Although sarcopenia, frailty status, and systemic inflammation are relatively well established as negative predictors of clinical outcomes in patients with HNC undergoing surgery [18,19,20,21,34,35], the value of these factors to predict patients with OSCC undergoing adjuvant CRT is unclear. The present study analyzed 175 patients with OSCC, among which 64% (*n* = 112) had sarcopenia undergoing adjuvant CRT. We found sarcopenia to be significantly associated with CIA. Moreover, sarcopenia and PLR were independent risk factors for clinical variables in CIA. At three years after diagnosis, initial advanced stage, sarcopenia, and PLR were independent risk factors for shorter survival. These results highlight that pre-CRT sarcopenia and high PLR represent risks for complications and poor survival, in addition to advanced tumor stage. The prevalence of sarcopenia in our cohort (64%) is consistent with the previously published prevalence [6]. Many studies have demonstrated that sarcopenia is associated with increased chemotherapy dose-limiting toxicity [9], CRT toxicity [7,8,9,36], and radiation treatment breaks [7,9]. However, an association between CRT-related hematologic toxicity and sarcopenia has not been demonstrated in OSCC. Based on our results, sarcopenia was not associated with FN or infection. Posner et al. [37] reported that FN more frequently occurred in patients with HNC who received TPF than those who received PF. Nishikawa et al. [12] reported a correlation between sarcopenia and grade 1–2 radiation-induced toxicities, such as xerostomia and dysgeusia, in patients with HNC; however, that study included only 39 patients. Other studies focused on symptomatic toxicity, including dermatitis, mucositis xerostomia, dysgeusia, and hypothyroidism, but none found an association between sarcopenia and CRT-related acute hematologic toxicities. Here, we collected hematologic toxicity only because symptomatic toxicity may have been overlooked in medical records.

Systemic inflammatory response is associated with the development of anemia [38] and sarcopenia in cancer [39]. Since disease-related blood loss can be encountered in OSCC, CRT can be immunosuppressive, inhibiting erythropoiesis and gastrointestinal mucositis, resulting in further nutritional status deterioration. Chronic malnutrition may strengthen anemia severity, such as via folic acid and iron insufficiency [40]; this may also be partly caused by alcohol consumption in most of our patients. Consistent with a previous study [10,36], sarcopenia may be a more accurate marker for nutritional status than BMI. However, the impact of sarcopenia on CIA in patients with OSCC remains unclear; additional investigations are necessary. Moreover, we found that PLR was a predictor of CIA. Thrombocytosis can result from reactive processes such as acute blood loss, infections, and iron deficiency anemia [40,41]. However, further research is still required to show an effect between PLR and the cause of anemia during CRT in patients with OSCC. Many studies have investigated the association between high PLR and survival in patients with oral cancer [34]. Cancer cells induce platelet activation, and activated platelets influence tumor growth, angiogenesis, and metastasis. Therefore, platelets closely link inflammation with cancer progression [41]. In this study, we defined the cutoff value of PLR as 385, which is higher than that reported in other OSCC studies [34]. In our population, the baseline characteristics of smoking, drinking alcohol, and betel nut chewing reflect cumulative chronic inflammation and induce carcinogenesis; tobacco and alcohol cause DNA damage, inducing mutations and potentially altering the tumor immune microenvironment [42]. Therefore, inflammatory cells might be more strongly associated with tumor development in OSCC than at other sites. In our cohort, we did not find the systemic inflammation status predictive of DFS, nor did we find traditional clinicopathological characteristics PNI, LVI, and ECS to correlate with survival.

Despite optimized diagnosis and treatment, recurrence or metastasis develops in more than 65% of patients with HNC [42]. Additionally, we did not examine the effects of sarcopenia on treatment tolerance, such as treatment interruption and dose-limiting during CRT. On the other hand, all subjects received adjuvant CRT, indicating that they were at high risk of locoregional recurrence. These reasons may have caused difficulties in applying these indices retrospectively in our cohort as predictors of DFS. Studies have demonstrated a significant negative relationship between frailty and perioperative outcomes in HNC, including mortality, complications, longer hospital stay, and readmission rate [18,19,20,21]. In our dataset, there was no significant difference in patients with or without frailty in terms of CRT or survival. Rittberg et al. [43] found that mFI-11-defined frailty in patients with advanced pancreatic cancer receiving first-line palliative chemotherapy was not associated with chemotherapy toxicity, disease response, or survival. Previous studies demonstrated that pre-frailty and frailty were high in older adults with cancer, up to 42% and 43%, respectively [44]. In contrast, our study had a median age of 53.9 years, and 32.6% and 14.9% of patients were categorized as pre-frailty and frailty, respectively. The mFI-5 findings in our study may be influenced by demographics or treatment modality and may not be readily compared with the results of other investigations. A recent systematic review evaluating the association of functional or cognitive impairment, social conditions, and frailty with adverse results in patients with HNC failed to find any longitudinal studies assessing the association of frailty with adverse postoperative outcomes [45]. In addition, patients undergoing surgery followed by CRT may exhibit a less frail physical phenotype of frailty. The concept of frailty is evolving, and there remains no consensus on its use in clinical practice. Our study helps confirm the impact of sarcopenia on CIA and three-year survival, but our measure of frailty status was not correlated with these results.

The study also has limitations. First, many patients were excluded because of insufficient available images for SMI analysis, which might have led to selection bias. We measured SMA at C3 on the CT images because most of the pretreatment images were captured via CT and not through magnetic resonance imaging (MRI). Relative to CT, MRI offers specific image qualities for the differentiation of body composition changes. CT and MRI for the skeletal muscle at C3 can be used interchangeably in cases of sarcopenia [46]. Despite the use of the C3 level that is readily available with the prognostic value of sarcopenia in HNC patients, the assessment of the sternocleidomastoid muscles on CT images in patients with advanced OSCC can still be impaired by the tumor infiltration of lymphadenopathies. In the current study, patients with resectable OSCC usually presented without supra-notch invasion. Accordingly, the assessment of masticatory muscles at the mandibular notch level will not be impaired by the presence of primary tumor invasion or metastatic lymphadenopathy. Further large-scale studies are needed to investigate the optimal level for diagnosing sarcopenia on head and neck CT/MRI images. Second, the study was retrospective, and bias generated from the study design is possible. Additionally, in patients who developed surgery-related complications, adjuvant therapy is often delayed or limited. A significant number of patients were involved in the EORTC 22,931 trial [47]; nearly three-quarters of them met the inclusion criteria for adjuvant therapy, but only 44% of them received it. Our retrospective study has a small number of patients and is thus subject to selection bias and confounding. Extensive cohort studies are needed to investigate patients undergoing all treatment modalities and to analyze the subgroups of those undergoing adjuvant CRT for the increased sarcopenia and m-FI predictor values of statistical power. Finally, our results are based on a single medical center, and our calculated cutoff values cannot be applied in a prospective study. Future multicenter studies are warranted to fully understand factors that will improve the clinical outcomes of patients with OSCC and sarcopenia who are undergoing treatment.

## 5. Conclusions

Sarcopenia and high PLR are significant predictors of CIA and reduced three-year OS in patients with OSCC under adjuvant CRT. Both CT images and hematologic markers to predict sarcopenia and high PLR, respectively, are readily available, reproducible, and inexpensive in clinical evaluations. Additionally, using a novel comprehensive assessment would be a useful personalized prognostic tool for patients with OSCC, which may improve clinical outcomes by addressing patients’ specific needs.

## Figures and Tables

**Table 1:** Patient characteristics classified by sarcopenia before adjuvant chemoradiotherapy.

Variable	Total (*n* = 175)	Sarcopenia (*n* = 112)	No Sarcopenia (*n* = 63)	*p*
Age, years				
Mean (SD)	53.9 (9.9)	54 (9.5)	54 (10.4)	0.384
Sex, n (%)				
Male	151 (86.3)	112 (100)	39 (61.9)	<0.001
Female	24 (13.7)	0 (0)	24 (38.1)	
Alcohol, n (%)				
Never drinker	40 (22.9)	19 (16.9)	21 (33.3)	0.031
Ever drinker	135 (77.1)	93 (83.1)	42 (66.7)	
Smoking, n (%)				
Never drinker	34 (19.4)	22 (19.6)	11 (17.4)	0.592
Ever drinker	141 (80.6)	90 (80.4)	52 (82.6)	
Betel nut, n (%)				
Never chewing	35 (20.0)	18 (16.1)	17 (26.9)	0.101
Ever chewing	140 (80.0)	94 (83.9)	46 (73.1)	
mFI-5				
0	92 (52.5)	52 (46.4)	38 (60.3)	0.031
1	57 (32.6)	37 (33.0)	20 (31.8)	
≥2	26 (14.9)	23 (20.6)	5 (7.9)	
Cancer site				
Buccal	58 (33.1)	37 (33.0)	21 (33.3)	0.080
Lower gum	27 (15.4)	17 (15.1)	10 (15.9)	
Tongue	59 (33.7)	33 (29.4)	26 (41.3)	
Other sites	31 (17.8)	25 (22.5)	6 (9.5)	
Clinical Stage				
I	16 (9.1)	9 (8.0)	7 (11.1)	0.624
II	38 (21.7)	23 (20.5)	15 (23.8)	
III	10 (5.7)	5 (4.5)	5 (7.9)	
IV	111 (63.5)	75 (67.0)	36 (57.2)	
PNI				
Negative	79 (45.1)	50 (44.6)	29 (46.0)	0.947
Positive	96 (54.9)	62 (55.4)	34 (54.0)	
LVI				
Negative	80 (45.7)	53 (47.3)	27 (42.9)	0.451
Positive	95 (54.3)	59 (52.7)	36 (57.1)	
ECS				
Negative	130 (74.3)	82 (73.2)	48 (76.2)	0.702
Positive	45 (25.7)	30 (26.8)	15 (23.8)	
BMI, kg/m^2^				
Mean (SD)	25.2 (5.3)	23.6 (3.4)	28.1 (5.5)	<0.001
Albumin				
Median (IQR)	3.4 (3.1–3.7)	3.4 (3.1–3.7)	3.4 (3.1–3.7)	0.610
SMI, cm^2^/m^2^				
Mean ± SD	42.9 (8.8)	38.8 (5.7)	49.6 (8.1)	<0.001
Hemoglobin				
Mean ± SD	11.4 (1.9)	11.5 (1.9)	11.0 (1.6)	0.754
NLR				
Median (IQR)	3.4 (2.2–5.3)	3.9 (2.2–6.6)	3.3 (2.2–4.8)	0.072
PLR				
Median (IQR)	223.2 (142.7–341.4)	228.7 (147.8–347.3)	218.2 (142.6–326.6)	0.652

SD, standard deviation; IQR, interquartile range; mFI-5, five-item modified frailty index; PNI, perineural invasion; LVI, lymph vascular invasion; ECS, extracapsular spread; BMI, body mass index; SMI, skeletal mass index; NLR, neutrophil/lymphocyte ratio; PLR, platelet/lymphocyte ratio.

**Table 2:** Chemoradiotherapy-related toxicities according to sarcopenia and modified frailty index.

Toxicities	Sarcopenia (*n* = 112)	No Sarcopenia (*n* = 63)	*p*	mFI-5 ≥ 1 (*n* = 83)	mFI-5 = 0 (*n* = 92)	*p*
Anemia n (%)						
Grade 0–2	92 (82.1)	59 (93.6)	0.021	71 (85.5)	80 (86.6)	0.841
Grade 3–4	20 (17.9)	4 (6.4)		12 (14.5)	12 (13.4)	
Febrile neutropenia n (%)						
Grade 0–2	104 (92.9)	61 (96.8)	0.110	80 (96.4)	87 (94.5)	0.384
Grade 3-4	8 (7.1)	2 (3.2)		3 (3.6)	5 (5.5)	
Infection n (%)						
Grade 0–2	95 (84.8)	57 (90.5)	0.181	72 (86.7)	80 (86.9)	0.605
Grade 3–4	17 (15.2)	6 (9.5)		11 (13.3)	12 (13.1)	

mFI-5, five-item modified frailty index.

**Table 3:** Logistic regression of clinical variables in chemoradiotherapy-related toxicities outcomes.

				Univariate						Multivariate		
	Anemia			Febrile Neutropenia			Infection			Anemia		
	OR	95% CI	*p*	OR	95% CI	*p*	OR	95% CI	*p*	OR	95% CI	*p*
Age (≤65 vs. >65)	0.921	(0.514–1.528)	0.780	0.921	(0.524–1.582)	0.781	1.625	(0.75–3.62)	0.201			
Stage (I–II vs. III–IV)	0.918	(0.325–2.703)	0.971	1.612	(0.332–8.090)	0.554	2.118	(0.70–6.79)	0.172			
Sarcopenia (no vs. yes)	1.739	(1.931–3.107)	0.040	0.835	(0.567–1.471)	0.532	2.024	(0.71–5.82)	0.187	1.871	(1.051–3.320)	0.040
Hb (>11 vs. ≤11)	3.326	(1.171–9.615)	0.021	1.217	(0.276–5.610)	0.805	3.010	(1.16–7.77)	0.023	2.821	(0.925–8.208)	0.058
NLR (≤3.4 vs. >3.4)	3.622	(1.278–10.02)	0.011	0.835	(0.116–4.529)	0.853	1.714	(0.68–4.46)	0.241			
PLR (≤385 vs. >385)	2.018	(0.616–6.448)	0.204	0.922	(0.542–1.835)	0.781	1.349	(0.49–4.49)	0.473	3.102	(1.017–8.912)	0.033

OR, odds ratio; CI, confidence interval; Hb, hemoglobin; NLR, neutrophil/lymphocyte ratio; PLR, platelet/lymphocyte ratio.

**Table 4:** Univariate and multivariate analyses of clinical variables in relation to three-year overall survival.

	Overall Survival						Disease-Free Survival		
	Univariate			Multivariate			Univariate		
	HR	95% CI	*p*	HR	95% CI	*p*	HR	95% CI	*p*
Age	0.990	(0.861–1.622)	0.918				0.972	(0.915–1.215)	0.072
Stage (I–II vs. III–IV)	2.352	(1.415–3.180)	<0.001	1.820	(1.201–3.323)	0.042	1.464	(0.812–2.670)	1.195
PNI (negative vs. positive)	1.631	(1.104–2.423)	0.011	1.114	(0.713–1.779)	0.542	1.163	(0.925–2.580)	0.061
LVI (negative vs. positive)	1.172	(0.709–1.731)	0.410				1.513	(0.819–2.460)	0.112
ECS (negative vs. positive)	1.727	(1.122–2.624)	0.014	1.505	(0.971–2.461)	0.068	1.125	(0.710–2.204)	0.443
mFI (0 vs. ≥1)	1.273	(0.781–2.108)	0.321				1.718	(0.921–3.590)	0.692
BMI (≥18.5 vs. <18.5 kg/m^2^)	2.005	(0.819–4.710)	0.082				0.823	(0.471–1.487)	0.543
Sarcopenia (no vs. yes)	1.745	(1.123–2.627)	0.016	1.643	(1.202–2.670)	0.034	2.623	(0.912–4.570)	0.532
Hb (≤11 vs. >11)	0.906	(0.48–2.411)	0.896				0.619	(0.317–1.249)	0.251
NLR (≤3.4 vs. >3.4)	1.793	(1.116–2.176)	<0.001	1.006	(0.529–1.772)	0.974	1.607	(0.732–3.540)	0.232
PLR (≤385 vs. >385)	2.620	(1.610–4.213)	<0.001	1.983	(1.206–2.553)	0.033	2.205	(0.876–2.182)	0.748

HR, hazard ratio; CI, confidence interval; PNI, perineural invasion; LVI, lymphovascular invasion; ECS, extracapsular spread; mFI-5, five-item modified frailty index; BMI, body mass index; Hb, hemoglobin; NLR, neutrophil/lymphocyte ratio; PLR, platelet/lymphocyte ratio.

## Data Availability

The data presented in this study are available on request from the corresponding author. The data are not publicly available due to the privacy and ethical restrictions.

## References

[1] Bray F., Ferlay J., Soerjomataram I., Siegel R.L., Torre L.A., Jemal A. (2018). Global cancer statistics 2018: GLOBOCAN estimates of incidence and mortality worldwide for 36 cancers in 185 countries. CA Cancer J. Clin..

[2] Pfister D.G., Spencer S., Adelstein D., Adkins D., Anzai Y., Brizel D.M., Bruce J.Y., Bruce P.M., Caudell J.Y., Cmelak T. (2020). Head and Neck Cancers, Version 2.2020, NCCN Clinical Practice Guidelines in Oncology. J. Natl. Compr. Cancer Netw..

[3] Kim D., Li R. (2019). Contemporary Treatment of Locally Advanced Oral Cancer. Curr. Treat. Options Oncol..

[4] Cramer J.D., Johnson J.T., Nilsen M.L. (2018). Pain in Head and Neck Cancer Survivors: Prevalence, Predictors, and Quality-of-Life Impact. Otolaryngol. Head Neck Surg..

[5] Santilli V., Bernetti A., Mangone M., Paoloni M. (2014). Clinical definition of sarcopenia. Clin. Cases Miner. Bone Metab..

[6] Grossberg A.J., Chamchod S., Fuller C.D., Mohamed A.S., Heukelom J., Eichelberger H., Kantor M.E., Hutcheson K.A., Gunn G.B., Garden A.S. (2016). Association of Body Composition with Survival and Locoregional Control of Radiotherapy-Treated Head and Neck Squamous Cell Carcinoma. JAMA Oncol..

[7] Ganju R.G., Morse R., Hoover A., TenNapel M., Lominska C.E. (2019). The impact of sarcopenia on tolerance of radiation and outcome in patients with head and neck cancer receiving chemoradiation. Radiother. Oncol..

[8] Wong A., Zhu D., Kraus D., Tham T. (2021). Radiologically Defined Sarcopenia Affects Survival in Head and Neck Cancer: A Meta-Analysis. Laryngoscope.

[9] Wendrich A.W., Swartz J.E., Bril S.I., Wegner I., de Graeff A., Smid E.J., Pothen A.J. (2017). Low skeletal muscle mass is a predictive factor for chemotherapy dose-limiting toxicity in patients with locally advanced head and neck cancer. Oral Oncol..

[10] Achim V., Bash J., Mowery A., Guimaraes A.R., Li R., Schindler J., Wax M., Andersen P., Clayburghet D. (2017). Prognostic Indication of Sarcopenia for Wound Complication After Total Laryngectomy. JAMA Otolaryngol. Head Neck Surg..

[11] Bril S.I., Pezier T.F., Tijink B.M., Janssen L.M., Braunius W.W., de Bree R. (2019). Preoperative low skeletal muscle mass as a risk factor for pharyngocutaneous fistula and decreased overall survival in patients undergoing total laryngectomy. Head Neck.

[12] Nishikawa D., Hanai N., Suzuki H., Koide Y., Beppu S., Hasegawa Y. (2018). The Impact of Skeletal Muscle Depletion on Head and Neck Squamous Cell Carcinoma. ORL J. Otorhinolaryngol. Relat. Spec..

[13] Ueno H., Hawrylowicz C.M., Banchereau J. (2007). Immunological intervention in human diseases. J. Transl. Med..

[14] Szilasi Z., Jósa V., Zrubka Z., Mezei T., Vass T., Merkel K., Helfferich F., Baranyai Z. (2020). Neutrophil-To-Lymphocyte and Platelet-To-Lymphocyte Ratios as Prognostic Markers of Survival in Patients with Head and Neck Tumours—Results of a Retrospective Multicentric Study. Int. J. Environ. Res. Public Health.

[15] De Vries N.M., Staal J.B., van Ravensberg C.D., Hobbelen J.S., Olde Rikkert M.G., Nijhuis-van der Sanden M.W. (2011). Outcome instruments to measure frailty: A systematic review. Ageing Res. Rev..

[16] Dodds R., Sayer A.A. (2016). Sarcopenia and frailty: New challenges for clinical practice. Clin. Med..

[17] Ethun C.G., Bilen M.A., Jani A.B., Maithel S.K., Ogan K., Master V.A. (2017). Frailty and cancer: Implications for oncology surgery, medical oncology, and radiation oncology. CA Cancer J. Clin..

[18] Goldstein D.P., Sklar M.C., de Almeida J.R., Gilbert R., Gullane P., Irish J., Brown D., Higgins K., Enepekides D., Xu W. (2020). Frailty as a predictor of outcomes in patients undergoing head and neck cancer surgery. Laryngoscope.

[19] Abt N.B., Richmon J.D., Koch W.M., Eisele D.W., Agrawal N. (2016). Assessment of the Predictive Value of the Modified Frailty Index for Clavien-Dindo Grade IV Critical Care Complications in Major Head and Neck Cancer Operations. JAMA Otolaryngol. Head Neck Surg..

[20] Adams P., Ghanem T., Stachler R., Hall F., Velanovich V., Rubinfeld I. (2013). Frailty as a predictor of morbidity and mortality in inpatient head and neck surgery. JAMA Otolaryngol. Head Neck Surg..

[21] Wachal B., Johnson M., Burchell A., Sayles H., Rieke K., Lindau R., Lydiatt W., Panwaret A. (2017). Association of Modified Frailty Index Score With Perioperative Risk for Patients Undergoing Total Laryngectomy. JAMA Otolaryngol. Head Neck Surg..

[22] Liu S.H., Chao K.S., Leu Y.S., Lee J.C., Liu C.J., Huang Y.C., Chang Y.F., Chen W.H., Tsai J.T., Chen Y.J. (2015). Guideline and preliminary clinical practice results for dose specification and target delineation for postoperative radiotherapy for oral cavity cancer. Head Neck.

[23] National Cancer Institute (NCI) Common Terminology Criteria for Adverse Events (CTCAE) v5.0 U.S. https://ctep.cancer.gov/protocoldevelopment/electronic_applications/ctc.htm#ctc_50.

[24] Heymsfield S.B., Wang Z., Baumgartner R.N., Ross R. (1997). Human body composition: Advances in models and methods. Annu. Rev. Nutr..

[25] Swartz J.E., Pothen A.J., Wegner I., Smid E.J., Swart K.M., de Bree R., Leenen L.P.H., Grolman W. (2016). Feasibility of using head and neck CT imaging to assess skeletal muscle mass in head and neck cancer patients. Oral Oncol..

[26] Ufuk F., Herek D., Yüksel D. (2019). Diagnosis of Sarcopenia in Head and Neck Computed Tomography: Cervical Muscle Mass as a Strong Indicator of Sarcopenia. Clin. Exp. Otorhinolaryngol..

[27] Van Rijn-Dekker M.I., van den Bosch L., van den Hoek J.G.M., Bijl H.P., van Aken E.S.M., van der Hoorn A., Oosting S.F., Halmos H.G., Witjes M.J.H., van der Laan H.P. (2020). Impact of sarcopenia on survival and late toxicity in head and neck cancer patients treated with radiotherapy. Radiother. Oncol..

[28] Rosset A., Spadola L., Ratib O. (2004). OsiriX: An open-source software for navigating in multidimensional DICOM images. J. Digit. Imaging.

[29] Velanovich V., Antoine H., Swartz A., Peters D., Rubinfeld I. (2013). Accumulating deficits model of frailty and postoperative mortality and morbidity: Its application to a national database. J. Surg. Res..

[30] Hermiz S.J.R., Lauzon S., Brown G., Herrera F.A. (2021). Use of a 5-Item Modified Frailty Index for Risk Stratification in Patients Undergoing Breast Reconstruction. Ann. Plast Surg..

[31] Subramaniam S., Aalberg J.J., Soriano R.P., Divino C.M. (2018). New 5-Factor Modified Frailty Index Using American College of Surgeons NSQIP Data. J. Am. Coll. Surg..

[32] Chimukangara M., Helm M.C., Frelich M.J., Bosler M.E., Rein L.E., Szabo A., Gould J.C. (2017). A 5-item frailty index based on NSQIP data correlates with outcomes following paraesophageal hernia repair. Surg. Endosc..

[33] Youden W.J. (1950). Index for rating diagnostic tests. Cancer.

[34] Zhang Y., Zheng L., Quan L., Du L. (2021). Prognostic role of platelet-to-lymphocyte ratio in oral cancer: A meta-analysis. J. Oral Pathol. Med..

[35] Lee J., Liu S.H., Dai K.Y., Huang Y.M., Li C., Chen J.C., Leu Y.S., Liu C.J., Chen Y.J. (2021). Sarcopenia and Systemic Inflammation Synergistically Impact Survival in Oral Cavity Cancer. Laryngoscope.

[36] Huang C.H., Lue K.H., Hsieh T.C., Liu S.H., Wang T.F., Peng T.C. (2020). Association between sarcopenia and clinical outcomes in patients with esophageal cancer under neoadjuvant therapy. Anticancer. Res..

[37] Posner M.R., Hershock D.M., Blajman C.R., Mickiewicz E., Winquist E., Gorbounova V., Tjulandin S., Shin D.M., Cullen K., Ervin T.J. (2007). Cisplatin and fluorouracil alone or with docetaxel in head and neck cancer. N. Engl. J. Med..

[38] Ganz T. (2019). Anemia of Inflammation. N. Engl. J. Med..

[39] Chindapasirt J. (2015). Sarcopenia in Cancer Patients. Asian Pac. J. Cancer Prev..

[40] Mhadgut H., Galadima H., Tahhan H.R. (2018). Thrombocytosis in iron deficiency anemia. Blood.

[41] Franco A.T., Corken A., Ware J. (2015). Platelets at the interface of thrombosis, inflammation, and cancer. Blood.

[42] Chow L.Q.M. (2020). Head and Neck Cancer. N. Engl. J. Med..

[43] Rittberg R., Zhang H., Lambert P., Kudlovich R., Kim C.A., Dawe D.E. (2021). Utility of the modified frailty index in predicting toxicity and cancer outcomes for older adults with advanced pancreatic cancer receiving first-line palliative chemotherapy. J. Geriatr. Oncol..

[44] Handforth C., Clegg A., Young C., Simpkins S., Seymour M.T., Selby P.J., Young J. (2015). The prevalence and outcomes of frailty in older cancer patients: A systematic review. Ann. Oncol.

[45] Van Deudekom F.J., Schimberg A.S., Kallenberg M.H., Slingerland M., van der Velden L.A., Mooijaart S.P. (2017). Functional and cognitive impairment, social environment, frailty and adverse health outcomes in older patients with head and neck cancer, a systematic review. Oral Oncol..

[46] Zwart A.T., Becker J.N., Lamers M.J., Dierckx R.A.J.O., de Bock G.H., Halmos G.B., van der Hoorn A. (2021). Skeletal muscle mass and sarcopenia can be determined with 1.5-T and 3-T neck MRI scans, in the event that no neck CT scan is performed. Eur. Radiol..

[47] Bernier J., Domenge C., Ozsahin M., Matuszewska K., Lefèbvre J.L., Greiner R.H., Giralt J., Maingon P., Rol land F., Bolla M. (2004). Postoperative irradiation with or without concomitant chemotherapy for locally advanced head and neck cancer. N. Engl. J. Med..

